# Surgical Outcomes and Prognostic Factors of G3 Pancreatic Neuroendocrine Carcinomas: A Consecutive Analysis Based on Previous Study Results

**DOI:** 10.3390/jcm11113176

**Published:** 2022-06-02

**Authors:** Xinmei Luo, Min Yang, Bole Tian, Xubao Liu, Kaiti Duan, Yi Zhang

**Affiliations:** 1 Department of Health Management Center, West China Hospital of Sichuan University, Chengdu 610041, China; luoxinmei888@163.com.cn; 2 Department of Pediatric Surgery, West China Hospital of Sichuan University, Chengdu 610041, China; hx2014bsym@163.com.cn; 3 Department of Pancreatic Surgery, West China Hospital of Sichuan University, Chengdu 610041, China; hxtbl0338@126.com (B.T.); xbliu@medmail.com.cn (X.L.); 4 Frank H. Netter MD School of Medicine, North Haven, CT 06473, USA; kaiti.duan@quinnipiac.edu

**Keywords:** pancreatic neuroendocrine carcinomas, WHO, grading, resection, prognosis

## Abstract

In 2017, the World Health Organization (WHO) officially defined pancreatic neuroendocrine neoplasms into well-differentiated tumors, namely G1/G2/G3 pancreatic neuroendocrine tumors, and poorly differentiated carcinomas referring to G3 pancreatic neuroendocrine carcinomas (p-NECs). However, the surgical outcomes and prognostic factors of G3 p-NECs are still unclear. **Methods:** We retrospectively collected and analyzed the data of eligible patients with G3 p-NECs defined by the WHO 2017 grading classification. **Results:** We eventually identified 120 patients with G3 p-NECs, including 72 females and 48 males, with a median age of 53 y. The 3-year overall survival (OS) of G3 p-NECs by Kaplan–Meier method was 37.3%. The 3-year OS for functional G3 p-NECs was 57.4%, which was statistically longer than 23.0% of non-functional ones (*p* = 0.002). Patients with surgical resection presented a significantly better 3-year OS than those with palliative operation (43.3% vs. 13.1%; *p* < 0.001). The 3-year OS for Stage Ⅰ, Stage Ⅱ, Stage Ⅲ, and Stage Ⅳ was 87.1%, 56.5%, 12.9%, and not applicable, respectively (*p* < 0.001). We demonstrated in a Cox regression model that palliative operation (*p* = 0.013), vascular infiltration (*p* = 0.039), lymph node involvement (*p* = 0.024), and distant metastasis (*p* = 0.016) were independent predictors of poor outcome for patients with surgically treated G3 p-NECs. **Conclusion:** Our data in the present analysis indicated that patients with G3 p-NECs could significantly benefit from surgical resection. Meanwhile, vascular infiltration, lymph node involvement, and distant metastasis were independent predictors of poor outcome for these patients.

## 1. Introduction

Pancreatic neuroendocrine neoplasms (p-NENs), i.e., islet cell tumors, are a group of highly heterogeneous tumors with significantly different clinical features [1,2,3,4,5]. P-NENs comprise about 1% to 2% of all clinically detected pancreatic tumors, with an estimated annual worldwide incidence of 0.25 to 0.5 in 100,000 individuals [1,4,6]. However, p-NENs have been increasingly diagnosed during the past several decades, probably due to improvements in both clinicians’ awareness of this disease and the ability to detect localized and asymptomatic tumors by imaging modalities [1,6,7,8].

The first case of p-NENs was reported over 100 years ago [9], though we still find it difficult to classify p-NENs into prognostic groups for survival analysis due to their rarity and heterogeneity. In 2010, based on the well-known histological definitions of p-NENs in 2000 [10], the World Health Organization (WHO) classified p-NENs into Grading 1 (G1) pancreatic neuroendocrine tumors (p-NETs), G2 p-NETs, and G3 pancreatic neuroendocrine carcinomas (“G3 p-NECs”) [11], which was first introduced by the European Neuroendocrine Tumor Society (ENETS) [12]. Furthermore, in 2017, WHO officially separated p-NENs into two different groups, including well-differentiated tumors, namely G1/G2/G3 p-NETs, and poorly differentiated carcinomas referring to G3 p-NECs [13].

The WHO 2017 grading system for p-NENs aimed to improve the prediction of clinical outcomes and to help clinicians to select better therapeutic strategies for patient care and management [13]. Our previous research demonstrated that the WHO 2017 grading classification has made an important improvement on the WHO 2010 grading criteria because of its better ability to classify p-NENs into prognostic groups [14]. Nevertheless, the surgical outcomes and prognostic factors of the newly defined G3 p-NECs are still unclear. Therefore, with the results of our previous study [14], we here attempted to carry out an in-depth analysis of the clinical characteristics of G3 p-NECs. Moreover, we emphasized demonstrating the prognostic predictors for the survival of G3 p-NECs.

## 2. Materials and Methods

### 2.1. Patients Enrollment

This was a retrospective study referring to patients with G3 p-NECs undergoing surgical treatment between January 2002 and May 2020 in one of the largest medical institutes in China. We enrolled patients who were surgically treated, either by resection or biopsy, while those without any operation were excluded. With the agreement of the principles of Helsinki Declaration [15], the written informed consent of the present study was obtained on admission from all patients. Our research was approved by the Institutional Review Board and Ethics Committee of our hospital, as it was a consecutive analysis based on previous study results [14]. As before [14,16,17,18], the present analysis was performed according to tumor site in pancreas, tumor size, histopathology, and type of operation; demographic data included sex, age, and symptoms at presentation; treatment-related factors included date and type of operation, surgical complications, length of stay in hospital, and so on.

### 2.2. Tumor Features

In the present study, we defined G3 p-NECs as functional if patients presented symptoms related to hormone overproduction, such as insulinoma, gastrinoma, glucagonoma, etc., and nonfunctional if they did not. According to the documented definitions [10,14,19,20], poorly differentiated tumors manifest nodular or solid architecture lack of organoid traits, usually with high nucleocytoplasm ratio and multifocal or extensive tumor necrosis. In light of the WHO 2017 grading classification for p-NENs, G3 p-NECs were defined as having >20 mitoses per 10 high power fields (HPFs) or a Ki-67 proliferation index >20%, with poorly differentiated small cell or large cell features [13] All cases were staged according to the tumor-node-metastasis (TNM) system introduced by the American Joint Committee on Cancer (AJCC) 8th staging manual [21]. For enrolled patients, all surgical specimens from tumor tissues were re-stained with hematoxylin-eosin and immunohistochemical methods, which were microscopically reviewed by experienced pathologists in our institution. The histopathological features of all p-NECs were systematically documented in the prepared tabulations, as we performed in the previous study [14].

### 2.3. Statistical Analyses

In the present study, we report quantitative variables as means with standard deviation (SD) or medians and categorical variables as numbers with their frequencies as proportions (%). Similar to our previous studies [14,16,17,18], we conducted the follow-up by telephone, e-mail, mail, or outpatient clinic review between July 2019 and February 2021. Overall survival (OS) was calculated either as the time in months between the date of surgery and the date of death or last follow-up and presented as either median survival time (MST) or OS with a hazard ratio (HR) and 95% confidence intervals (CIs). We applied the Kaplan–Meier (K–M) method to generate the OS estimates and compared them by the log-rank test. Finally, we performed univariate and multivariate analysis in Cox regression proportional hazards model to demonstrate the prognostic predictors for the outcome of G3 p-NECs. Statistical analyses were performed using IBM SPSS 25.0 statistical software, which was defined as significant if the P value was less than 0.05.

## 3. Results

### 3.1. Baseline Demographics and Tumor Characteristics

As Table 1 presents, we identified 120 eligible patients with G3 p-NECs in this research. Our study cohort was composed of 72 females and 48 males, with a mean age at diagnosis of 50.2 ± 13.3 y and a median of 53 y (ranging from 14 y to 86 y). Most patients (84.2%) were diagnosed after the year of 2010 and most cases (79.2%) were solitary. The mean tumor diameter was 6.8 ± 3.5 cm, with a median of 5 cm (ranging from 1.5 cm to 13.5 cm). There were 66 tumors detected in the body and tail of the pancreas, while 54 were in the head and uncinate. In light of patients’ clinical manifestations and the tumors’ functional status, 50 patients presented as functional, in which insulinomas accounted for the majority (36 cases). As for 70 patients with nonfunctional G3 p-NECs, abdominal pain and distension was the main clinical manifestation of 46 patients, while abdominal mass and weight loss was that of 38 patients, with jaundice being that of 25 patients. Meanwhile, there were 37 patients with incidental diagnosis who might be detected by routine physical examinations.

Abdominal US, CT, and MRI were, respectively, performed in 94, 68, and 72 patients, whose positivity rate was 74.5%, 85.3%, and 83.3%. A total of 75 patients received postoperative medical therapy, including 24 cases with molecular targeting treatment and 51 with traditional platinum-based chemotherapy. The median Ki-67 positive index and mitotic rate of G3 p-NECs was respectively 62% (ranging from 23% to 90%) and 40 per 10 HPFs (ranging from 28 per 10 HPFs to 62 per 10 HPFs). For the functional group, the Ki-67 positive index ranged from 23% to 75%, with a median of 46%, while that of the nonfunctional ones ranged from 31% to 90%, with a median of 71%. In terms of the TNM staging system, there were 25 patients presenting with vascular infiltration, 55 cases with lymph node involvement, and 33 with distant metastasis, leading to a distribution of 22, 35, 30, and 33 patients, respectively, in Stage Ⅰ, Stage Ⅱ, Stage Ⅲ, and Stage Ⅳ.

### 3.2. Surgical Treatment and Postoperative Complication

As Table 2 presents, surgical resection was successfully performed for 94 patients, while a palliative operation was carried out for 26 patients. For patients with resections, 74 cases were of R0 status with both grossly and microscopically negative surgical margin, while 20 patients showed either grossly or microscopically positive surgical margin (i.e., R1/R2). Referring to the detailed surgical procedure, distal pancreatectomy (32.5%) and pancreaticoduodenectomy (30.8%) were the two most common approaches, followed by local resection of pancreatic tumor (referring to enucleation; 8.3%). A biopsy was performed for all patients with palliative operation (21.7%) in order to acquire the enough surgical specimens from tumor tissues to confirm the diagnosis of G3 p-NECs. The anesthesia grade from Ⅰ to Ⅴ by the American Society of Anesthesiologists was respectively evaluated in 14, 34, 45, 27, and 0 patients. There were 36 patients who experienced perioperative blood transfusion, with a mean volume of 420.5 ± 118.8 mL and a median of 400 mL (ranging from 100 mL to 1500 mL). The mean duration of operation was 202.4 ± 82.5 min, with a median of 180 min (ranging from 80 min to 510 min). A total of 42 patients had intensive care unit (ICU) in-hospital stays postoperatively, with a mean duration of 4.2 ± 1.8 d and a median of 3 d (ranging from 1 d to 10 d). The mean duration of postoperative and total in-hospital stay was, respectively, 12.4 ± 8.6 d and 21.2 ± 14.4 d, with a separate median of 9 d (ranging from 3 d to 36 d) and 11 d (ranging from 7 d to 52 d).

Of all the surgically treated patients with G3 p-NECs, 30 experienced postoperative complications, with a morbidity of 25.0% (Table 2). Pancreatic fistulas occurred in 21 patients, which was the most common postoperative complication (17.5%), followed by intra-abdominal infection (8.3%) and pulmonary infection (7.5%). Other complications, such as wound infection (4.2%), delayed gastric emptying (4.2%), intestinal obstruction (3.3%), intra-abdominal hemorrhage (2.5%), biliary fistula (1.7%), and intestinal fistula (1.7%), were uncommon. There was 1 in-hospital death caused by intra-abdominal hemorrhage, with a mortality of 0.8%. A total of 5 patients experienced reoperation (4.2%), including 2 cases for wound infection, 1 for pancreatic fistula, 1 for intra-abdominal hemorrhage, and 1 for intra-abdominal infection. All other postoperative complications could be treated well through non-operational therapies, such as appropriate medical treatments and unobstructed drainages.

### 3.3. Survival Estimates and Prognostic Analyses

**Figure 1:**
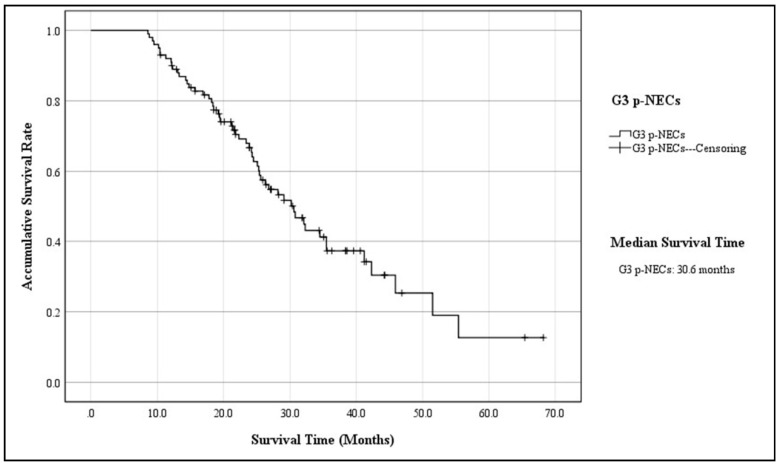
Kaplan–Meier estimates for the OS of G3 p-NECs.

**Figure 2:**
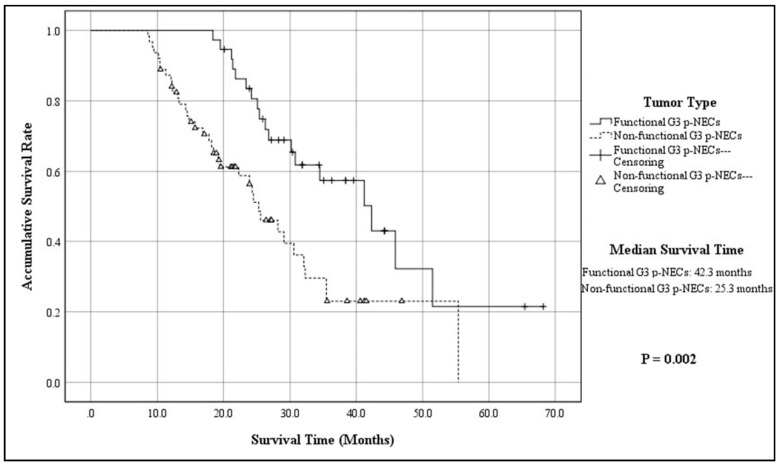
Kaplan–Meier estimates for the OS of G3 p-NECs, according to the tumor type.

**Figure 3:**
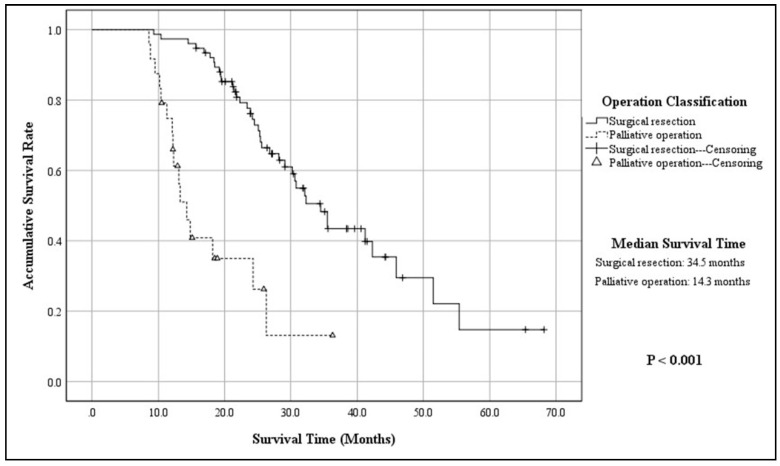
Kaplan–Meier estimates for the OS of G3 p-NECs, according to the operation classification.

**Figure 4:**
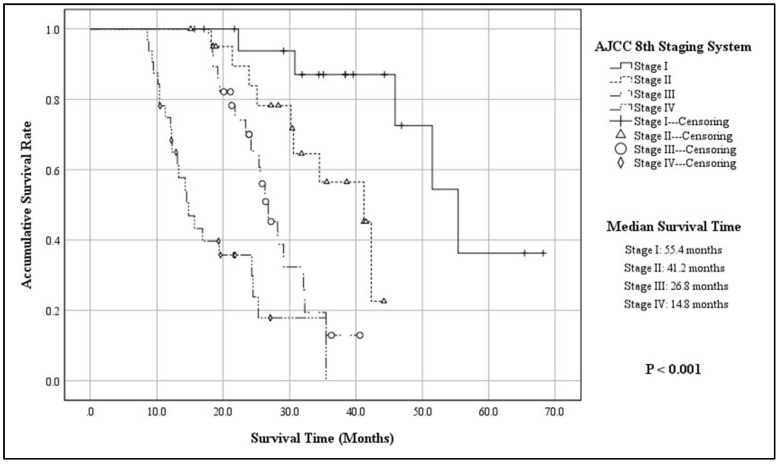
Kaplan–Meier estimates for the OS of G3 p-NECs, according to the AJCC 8th staging system.

The mean follow-up time of 100 patients was 48.8 ± 15.6 months, with a median of 56.8 months (ranging from 10.3 months to 176.4 months), while 20 patients were out of contact (16.7%). When the follow-up ended, there were 45 patients alive, whereas 55 were dead due to the progression of disease (55.0%). According to the K–M method, the accumulative 3-year OS of the entire cohort was 37.3%, with a MST of 30.6 months (95% CIs: 24.8–36.3; Figure 1). The 3-year OS and MST of functional G3 p-NECs were respectively 57.4% and 42.3 months (95% CIs: 30.5–54.1), while those of nonfunctional ones were 23.0% and 25.3 months (95% CIs: 20.8–29.7; *p* = 0.002; Figure 2). Patients with surgical resection obtained a 3-year OS of 43.4% and a MST of 34.5 months (95% CIs: 29.7–39.2), which was statistically better than that of patients with palliative operation (13.1%; 14.3 mons (95% CIs: 11.9–16.7); *p* < 0.001; Figure 3). The OS at 3 years for patients in Stage I, Stage II, Stage III, and Stage IV was, respectively, 87.1%, 56.5%, 12.9%, and not applicable, with a MST of 55.4 months (95% CIs: 45.3–65.4), 41.2 months (95% CIs: 34.6–47.7), 26.8 months (95% CIs: 23.6–29.9), and 14.8 months (95% CIs: 11.7–17.8). To be specific, survivals of patients in Stage I or Stage II were statistically better than those in Stage III (*p* < 0.001, *p* = 0.008, respectively) or Stage IV (*p* < 0.001, *p* < 0.001, respectively; Figure 4). Meanwhile, survival differences when comparing Stage I with Stage II or Stage III with Stage IV were also significant (*p* = 0.011, *p* = 0.001, respectively; Figure 4).

As Table 3 listed, sex, age, tumor site, incidental diagnosis, duration of operation, duration of postoperative in-hospital stay, ICU in-hospital stay, perioperative blood transfusion, and postoperative complication presented no notable differences in univariate analyses (*p* > 0.05). According to the subsequent multivariate analyses, tumor type, tumor diameter, anesthesia grade, surgical margin, and postoperative medical therapy were not notably significant (*p* > 0.05), while operation classification (*p* = 0.013), vascular infiltration (*p* = 0.039), lymph node involvement (*p* = 0.024), and distant metastasis (*p* = 0.016) were independent predictors for the prognosis of G3 p-NECs.

## 4. Discussion

P-NENs are a heterogeneous group of malignancies [1,2,3]. The grading classification based on mitotic counts and Ki-67 proliferation index by WHO in 2010 [11] has reflected great clinical value with widespread acceptance [22,23,24,25]. However, accumulated studies have demonstrated that those “G3 p-NECs” by the WHO 2010 grading system were morphologically and biologically heterogeneous, with different clinical-pathological features and long-term survivals [26,27,28,29,30]. Therefore, as reviewed by Julie et al. in their report [20], the heterogeneity of “G3 p-NECs” has promoted the emergence of the new WHO grading classification in 2017 [13], whose clinical value has just been validated by our studying team [14].

The present research was a consecutive analysis based on our previous report [14], because as a new sub-category of p-NENs, the surgical outcomes and prognostic factors of G3 p-NECs have not been comprehensively analyzed before. As reported [20,27,31], the clinical features of G3 p-NECs were very similar to typical pancreatic exocrine adenocarcinomas (p-EACs). Our analyses revealed that patient sex of G3 p-NECs had a slight female predominance (60%) with a median age of 53 y and that G3 p-NECs more frequently involved the body and tail of the pancreas (55%). These findings were basically in agreement with what was reported in our previous study [14]. Meanwhile, nonfunctional tumors accounted for most G3 p-NECs (70%), in which abdominal pain and distension, abdominal mass and weight loss, and jaundice were the main clinical presentations (38.3%, 31.7%, and 20.8% respectively), while incidental diagnosis was also obtained by physical examinations or others from 30.8% patients. Sorbye et al. reported that obstructive jaundice or nonspecific abdominal complaints might be the only signs or symptoms available to the suspicion of G3 p-NECs [32]. We here revealed that functional G3 p-NECs obtained a notably better survival than nonfunctional ones (57.4% vs. 23.0%; *p* = 0.002; Figure 2), probably due to earlier diagnosis based on clinical symptoms. However, tumor type still could not be a significant predictor for the prognosis of G3 p-NECs in the Cox regression model (*p* = 0.061; Table 3), as we have demonstrated [17].

Most G3 p-NECs were very mitotically active and cases with >40 to 50 mitoses per HPFs or Ki-67 proliferation index >50% were frequently observed [26,27,28,29]. Similarly, the median Ki-67 index and mitotic rate of the entire group were respectively 62% and 40 per 10 HPFs. As for clinical stage of G3 p-NECs, we previously demonstrated that the AJCC 8th TNM staging system originally applied to p-EACs was applicable for G3 p-NECs due to its better prognostic stratification and more accurate predicting ability [17]. According to our present analyses, we also succeeded in classifying G3 p-NECs into 4 groups with significantly different survivals by this staging system (*p* < 0.001; Figure 4).

G3 p-NECs could be treated by both surgical and medical therapy according to their clinical features, especially tumor grade and clinical stage [3,4,5,26,27,28,29]. In our present study, surgical resection was carried out for 94 patients with G3 p-NECs, in which distal pancreatectomy and pancreaticoduodenectomy were the two main procedures (32.5% and 30.8%; respectively), while palliative operation with biopsy was performed for 21.7% cases. As we proved in Table 3, operation classification was an independent predictor for the prognosis of G3 p-NECs (*p* = 0.013), in which patients could significantly benefit from surgical resection more than palliative operation (43.4% vs. 13.1%; *p* < 0.001; Figure 3). Moreover, patients with R0 surgical margin showed longer survival compared with those with R1/R2 margin (*p* = 0.012), while the surgical margin still failed to be proven as an independent predictor in the multivariate analyses (*p* = 0.092). Interestingly, we here had 10 cases of G3 p-NECs in which local resections of pancreatic tumor (referring to enucleation) were performed. We currently agree that a more radical approach for G3 p-NECs would be considered standard (identically to p-EACs). However, G3 p-NECs in this research were finally diagnosed by postoperative pathological examinations from the surgical specimens, which meant we did not know the neuroendocrine phenotype of the pancreatic lesion during operation. Moreover, enucleation of pancreatic tumor was carried out mainly in the early years when the biological behaviors of G3 p-NECs were not clear. It would be interesting to know the prognostic difference among distal pancreatic resection and pancreaticoduodenectomy with local resection. However, the power of this analysis would indeed be insufficient, due to the small number of cases with enucleation (only 10 cases). 

When the diagnosis of either “G3 p-NECs” by WHO 2010 grading classification or the present G3 p-NECs by WHO 2017 grading criteria was made by the postoperative pathological examinations, adjuvant therapy was routinely indicated in our hospital. However, drugs for the medical therapy varied over time, from the molecular targeted therapy at the beginning, such as sunitinib, everolimus, and octreotide, to the platinum-based drugs proposed by guidelines later [33], such as cisplatin and oxaliplatin. In the present study, we identified 75 patients who received postoperative medical therapy. Due to the small number of cases with each drug, we classified these patients into 24 cases with molecular targeting treatment and 51 with platinum-based chemotherapy. We found that patients could benefit from platinum-based chemotherapy, presenting a statistically longer survival than those with molecular targeting treatment (*p* = 0.037; Table 3), but postoperative medical therapy could not be a significant predictor for the outcome of G3 p-NECs (*p* = 0.184; Table 3), as we reported before [17].

Our study also had some limitations [14]. First, it was a retrospective study. Secondly, the accumulative OS was estimated by K–M methods. Then, our analysis derived from one single medical institution. Finally, we only enrolled patients who were surgically treated, either by resection or biopsy, while those without any operation were excluded. Therefore, a particular implication for G3 p-NECs, particularly those with metastatic disease at presentation might be unsuitable for any operation, given that surgery would not be considered as standard management for some patients. Moreover, surgery might not be strongly recommended from this case series since the better outcomes could be mainly related to lead time bias. With the above limitations, our present study still achieved the expected goal and will be of great value in guiding the treatment and prognosis of G3 p-NECs.

## 5. Conclusions

In sum, based on the studying results of our previous research, we carried out a consecutive analysis on the surgical outcomes and prognostic factors of G3 p-NECs in the present study. According to our demonstrations, G3 p-NECs could notably benefit from surgical resection, while vascular infiltration, lymph node involvement, and distant metastasis were independent predictors of poor prognosis for these patients.

## Figures and Tables

**Table 1:** Clinical characteristics of G3 p-NECs in the present study (N = 120).

Factor	Patients	
	No.	%
Patient sex		
Female	72	60.0
Male	48	40.0
Patient age at diagnosis, y		
Mean	50.2 ± 13.3	
Median (Range)	53 (14–86)	
Patient diagnostic period		
Before 2010	19	15.8
After 2010	101	84.2
Tumor number		
Solitary	95	79.2
Multiple	25	20.8
Tumor diameter, cm		
Mean	6.8 ± 3.5	
Median (Range)	5 (1.5–13.5)	
Tumor site		
Head and uncinate	54	45.0
Body and tail	66	55.0
Tumor functional status		
Functional tumors	50	41.7
Insulinoma	36	30.0
Others	14	11.7
Nonfunctional tumors	70	58.3
Abdominal pain and distension	46	38.3
Abdominal mass and weight loss	38	31.7
Jaundice	25	20.8
Incidental diagnosis	37	30.8
Preoperative imaging examinations		
US positive (N = 94)	70	74.5
CT positive (N = 68)	58	85.3
MRI positive (N = 72)	60	83.3
Postoperative medical therapy	75	62.5
Molecular targeting treatment	24	20.0
Traditional platinum-based chemotherapy	51	42.5
Ki-67 index, (%)		
Mean	55	
Median (Range)	62 (23–90)	
Mitotic rate, (per 10HPFs)		
Mean	38	
Median (Range)	40 (28–62)	
Vascular infiltration	25	20.8
Lymph node involvement	55	45.8
Distant metastasis	33	27.5
Tumor TNM staging system		
Stage Ⅰ	22	18.3
Stage Ⅱ	35	29.2
Stage Ⅲ	30	25.0
Stage Ⅳ	33	27.5
Patient prognosis		
Follow-up time, mons		
Mean	48.8 ± 15.6	
Median (Range)	56.8 (10.3–176.4)	
Out of contact	20	16.7
Dead at follow-up	55	55.0
Estimated 3-year OS		37.30%
MST, mons.		30.6

Abbreviations: G: grading; p-NECs: pancreatic neuroendocrine carcinomas; US: ultrasound; CT; computed tomography; MRI: magnetic resonance imaging; HPFs: high power fields; TNM: tumor-node-metastasis; OS: overall survival; MST: median survival time.

**Table 2:** Surgical treatment and postoperative complication of G3 p-NECs in the present study (N = 120).

Factor	Patients	
	No.	%
Operation classification		
Surgical resection	94	78.3
Palliative operation	26	21.7
Surgical margin (N = 94)		
R0	74	78.7
R1/R2	20	21.3
Surgical procedure		
Local resection of pancreatic tumor	10	8.3
Distal pancreatectomy	39	32.5
Pancreaticoduodenectomy	37	30.8
Biopsy	26	21.7
Others	8	6.7
Anesthesia grade by ASA		
Ⅰ	14	11.7
Ⅱ	34	28.3
Ⅲ	45	37.5
Ⅳ	27	22.5
Ⅴ	0	0
Volume of perioperative blood transfusion, ml	36	30.0
Mean	420.5 ± 118.8	
Median (Range)	400 (100–1500)	
Duration of operation, min		
Mean	202.4 ± 82.5	
Median (Range)	180 (80–510)	
Duration of ICU in-hospital stay, d	42	35.0
Mean	4.2 ± 1.8	
Median (Range)	3 (1–10)	
Duration of postoperative in-hospital stay, d		
Mean	12.4 ± 8.6	
Median (Range)	9 (3–36)	
Duration of total in-hospital stay, d		
Mean	21.2 ± 14.4	
Median (Range)	11 (7–52)	
Total in-hospital cost, RMB		
Mean	50,212.4 ± 21,208.6	
Median (Range)	56,450 (28,905–10,983)	
Postoperative complication	30	25.0
Pancreatic fistula	21	17.5
Intra-abdominal infection	10	8.3
Pulmonary infection	9	7.5
Wound infection	5	4.2
Delayed gastric emptying	5	4.2
Intestinal obstruction	4	3.3
Intra-abdominal hemorrhage	3	2.5
Biliary fistula	2	1.7
Intestinal fistula	2	1.7
In-hospital death	1	0.8
Reoperation	5	4.2
Wound infection	2	1.7
Pancreatic fistula	1	0.8
Intra-abdominal hemorrhage	1	0.8
Intra-abdominal infection	1	0.8

Abbreviations: G: grading; p-NECs: pancreatic neuroendocrine carcinomas; R: radical; ASA: American Society of Anesthesiologists; ICU: intensive care unit; RMB: renminbi.

**Table 3:** Univariate and multivariate analyses of factors influencing the prognosis of G3 p-NECs in the present study (N = 120).

Factor	Univariate Analysis		Multivariate Analysis	
	HR (95% CIs)	*p*	HR (95% CIs)	*p*
Sex				
Male ^A^				
Female	0.894 (0.554–2.113)	0.625		
Age, y				
<Median				
≥Median	1.541 (0.509–2.639)	0.091		
Tumor site				
Head and uncinate				
Body and tail	1.083 (0.516–1.522)	0.493		
Tumor type				
Functional				
Nonfunctional	1.725 (0.652–3.356)	**0.031**	0.914 (0.673–1.487)	0.619
Incidental diagnosis				
No				
Yes	1.003 (0.357–1.766)	0.213		
Tumor diameter				
<Median				
≥Median	1.863 (0.387–2.263)	**0.047**	0.557 (0.267–1.013)	0.652
Anesthesia grade				
Ⅰ/Ⅱ				
Ⅲ/Ⅳ/Ⅴ	1.554 (0.446–2.731)	**0.038**	0.791 (0.381–1.451)	0.443
Operation classification				
Resection				
Palliative	3.215 (0.379–8.236)	**<0.001**	1.493 (0.513–4.343)	0.013
Surgical margin				
R0				
R1/R2	1.813 (0.425–2.091)	**0.012**	1.113 (0.453–1.853)	0.092
Duration of operation				
<Median				
≥Median	1.345 (0.521–2.892)	0.113		
Duration of postoperative in-hospital stay				
<Median				
≥Median	1.115 (0.371–1.983)	0.305		
Perioperative blood transfusion				
No				
Yes	1.563 (0.476–2.093)	0.235		
ICU in-hospital stay				
No				
Yes	1.212 (0.674–1.814)	0.354		
Postoperative complication				
No				
Yes	1.315 (0.784–2.336)	0.549		
Postoperative medical therapy				
TPC				
MTT	1.925 (0.486–3.065)	**0.037**	1.094 (0.334–1.985)	0.184
Vascular infiltration				
No				
Yes	2.412 (0.731–6.126)	**<0.001**	5.232 (1.263–11.225)	**0.039**
Lymph node involvement				
No				
Yes	3.335 (0.982–8.426)	**0.029**	1.903 (0.329–5.013)	**0.024**
Distant metastasis				
No				
Yes	4.576 (0.775–12.435)	<0.001	2.493 (0.416–13.436)	**0.016**

^A^: The above related factor was regarded as a reference in Cox analysis. Abbreviation: G: grading; p-NECs: pancreatic neuroendocrine carcinomas; HR: hazard ratio; CIs: confidence intervals; R: radical; ICU: intensive care unit; TPC: traditional platinum-based chemotherapy; MTT: molecular targeting treatment.

## Data Availability

The processed data required to reproduce these findings cannot be shared at this time as the data also form part of an ongoing study.
